# A Comprehensive Review of the Main Lignan Components of Schisandra chinensis (North Wu Wei Zi) and Schisandra sphenanthera (South Wu Wei Zi) and the Lignan-Induced Drug-Drug Interactions Based on the Inhibition of Cytochrome P450 and P-Glycoprotein Activities

**DOI:** 10.3389/fphar.2022.816036

**Published:** 2022-03-11

**Authors:** Feng Zhang, Jianxiu Zhai, Nan Weng, Jie Gao, Jun Yin, Wansheng Chen

**Affiliations:** ^1^ Department of Pharmacy, Changzheng Hospital, Navl Medical University (Second Military Medical University), Shanghai, China; ^2^ Shanghai Key Laboratory for Pharmaceutical Metabolite Research, Shanghai, China; ^3^ School of Traditional Chinese Material, Shenyang Pharmaceutical University, Shenyang, China; ^4^ School of Pharmacy, Shanghai University of Traditional Chinese Medicine, Shanghai, China; ^5^ School of Pharmacy, Research and Development Center of Chinese Medicine Resources and Biotechnology, Institute of Chinese Materia Medica, Shanghai University of Traditional Chinese Medicine, Shanghai, China

**Keywords:** Schisandra sphenanthera, Schisandraceae, lignans, drug-drug interactions, ADME, *Schisandra chinensis*

## Abstract

Wu Wei Zi is the dried fruit of *Schisandra chinensis* (Turcz.) Baill. or *Schisandra sphenanthera* Rehd. et Wils. (family Magnoliaceae). As a homology of medicine and food, it has been widely used in China for thousands of years, to tonify the kidney, and ameliorate neurological, cardiovascular, liver, and gastrointestinal disorders. As its increasing health benefits and pharmacological value, many literatures have reported that the combination of Wu Wei Zi in patients has led to fluctuations in the blood level of the combined drug. Therefore, it is extremely important to evaluate its safety concern such as drug-drug interactions (DDIs) when patients are under the poly-therapeutic conditions. This review summarized the effects of Wu Wei Zi extract and its major lignan components on cytochrome P450 and P-glycoprotein activities, the change of which could induce metabolic DDIs. Our review also elaborated on the differences of the major lignan components of the two *Schisandra* species, as well as the absorption, distribution, metabolism, and elimination of the major lignans. In conclusion, these results would enhance our understanding of the DDI mechanisms involving Wu Wei Zi, and may potentially untangle some differing and conflicting results in the future.

## Introduction

“Wu Wei Zi” refers to the ripe fruits of two plants in the genus *Schisandra*, *S. chinensis* (Turcz.) Baill. (North Wu Wei Zi) or *S. sphenanthera* Rehd. et Wils (South Wu Wei Zi). It has been long used as nutritional and fatigue-fighting food supplement in China, Japan, Korea, and Russia. Records suggest that Russian hunters would eat Wu Wei Zi to alleviate fatigue, improve night vision, and replenish body fluids, while Ainu used Wu Wei Zi to protect against seasickness and cold weather (Panossian and Wikman, 2008). Both North Wu Wei Zi and South Wu Wei Zi were described with functions of astringency, reinforcing *qi* to generate body fluids, and tonifying the kidney to calm the mind (Chinese Pharmacopoeia Commission, 2020), along with hepatoprotective and anti-diabetic activities (Mu et al., 2006; Liu et al., 2017). Additionally, the two species also host unique properties. For instance, South Wu Wei Zi is more suitable for the treatment against chronic cough, mild breathlessness, and skin inflammation (Huyke et al., 2007), while North Wu Wei Zi has better cardiovascular-protective, neuroprotective, and tonic activities (Panossian and Wikman, 2008; Chun et al., 2014; Chen et al., 2015). More than 100 traditional Chinese medicine prescriptions composed of North Wu Wei Zi and South Wu Wei Zi are documented in the Pharmacopoeia of the People’s Republic of China (Ali et al., 2018), with various pharmacological activities. With their increasing consumption, it is extremely important to evaluate the safety concern such as drug-drug interactions (DDIs) when patients are under the poly-therapeutic conditions.

**FIGURE 1:**
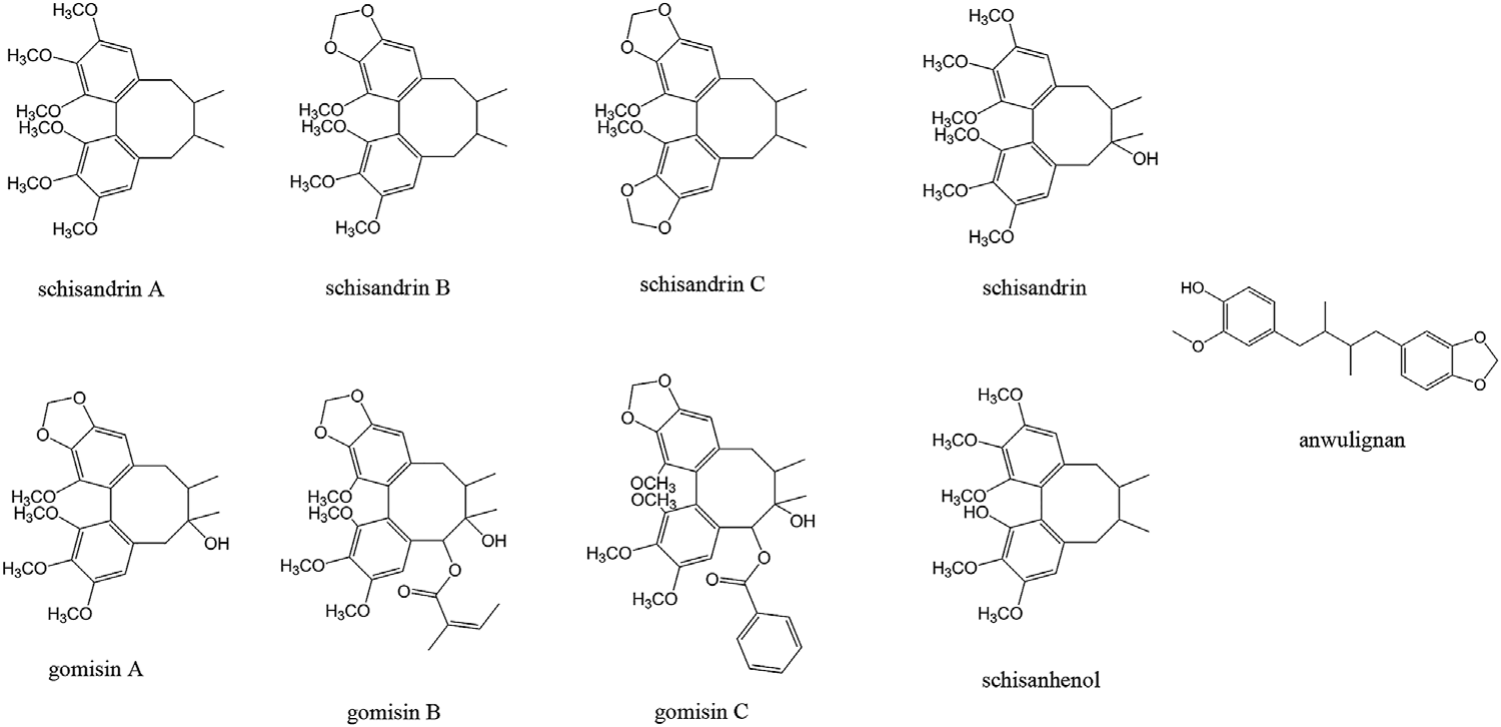
Structure of the major lignan components of Wu Wei Zi.

Recent studies revealed that both North Wu Wei Zi and South Wu Wei Zi are enriched in dibenzocyclooctadiene lignan components (Schisandra lignans, Figure 1), which are responsible for the major bioactivities of the two species (Sowndhararajan et al., 2018). However, the schisandra lignans are found to interfere with cytochrome P450 isoenzymes (CYPs) and P-glycoprotein (P-gp) activities, thus affecting the absorption, transportation, or metabolism of these or other drugs (Zhou et al., 2005; Qin et al., 2014a; Wang Cai-Ping et al., 2014), resulting metabolic DDIs. Depending on different medication circumstances, the “clinically relevant” DDI could lead to risk of adverse events or could be an advantageous for the therapy appropriateness processes (Palleria et al., 2020).

In recent years, the researchers paid more attention to the review of the botany, phytochemistry, chemical composition, in-depth *in vivo* pharmacological study, and clinical application of North Wu Wei Zi or South Wu Wei Zi (Szopa et al., 2017; Li et al., 2018; Huang et al., 2021), and only one review covered both North Wu Wei Zi and South Wu Wei Zi (Yang et al., 2022). However, little information was presented about their metabolic DDIs. In this review, we mainly focus on the main components of North Wu Wei Zi and South Wu Wei Zi, and identified CYP- and P-gp-interfering effects of the schisandra lignans, in order to better understand the quality control and potential DDIs of Wu Wei Zi products. By creating a full picture of the potential impacts of Wu Wei Zi, this review might facilitate investigations on DDIs for new chemical products and traditional Chinese medicines.

## Literature Search Strategy

Literature research was conducted in May 2021 for articles published from 2000 to 2021, without restriction to regions and publication type. Eligible literatures for inclusion are written in English or Chinese. Electronic databases such as PubMed, Web of Science, ScienceDirect, Springer, Wiley, China National Knowledge Infrastructure (CNKI) and WANFANG Data were searched by two authors independently. Detailed PubMed strategy was listed as follows: key words were composed of Wu Wei Zi/*Schisandra chinensis*/*Schisandra sphenanthera*/schisandra lignans and contents/pharmacokinetics/DDI/CYP/P-gp. Articles dealing with the geographical, history, botany, ethnomedicine were excluded. Most articles were from PubMed (85 articles), supplemented by Science Direct (2 articles), Web of Science (2 articles), CNKI (26 articles, 6 dissertations), WANFANG Data (4 articles).

## The Main Lignan Components of North Wu Wei Zi and South Wu Wei Zi

Schisandra lignans have a basic dibenzocyclooctadiene structure (Figure 1). They showed different contents in North Wu Wei Zi and South North Wu Wei Zi. The quantity and quality determinations of schisandra lignans to date are based on high-performance liquid chromatography (HPLC) methods such as HPLC-ultraviolet (UV), HPLC-capillary electrophoresis (CE), and HPLC-mass spectrometry (MS), or on gas chromatography-mass spectrometry (GC-MS) (Table 1). HPLC-UV has become the predominant method used to study Wu Wei Zi as there is a strong UV absorbance of schisandra lignans between 230 and 255 nm, resulting in high sensitivity and specificity (Zhang Hai et al., 2009).

**TABLE 1:** Contents of major schisandra lignans of Wu Wei Zi.

Type	Schisandrin A (mg/g)	Schisandrin B (mg/g)	Schisandrin C (mg/g)	Schisandrin (mg/g)	Gomisin A (mg/g)	Gomisin B (mg/g)	Gomisin C (mg/g)	Schisanhenol (mg/g)	Anwulignan (mg/g)	Det.	Ref.
Synonyms	deoxyschisandrin	*γ*-schisandrin	—	schisandrol A	schisandrol B	schisantherin B	schisantherin A	—	macelignan	—	—
Abbr.	Sch A	Sch B	Sch C	SCH	Gom A	Gom B	Gom C	—	—	—	—
North	—	8.00∼23.50	—	22.40∼37.50	7.40∼12.80	—	—	—	—	UV	Hu et al. (2014)
North	0.77∼2.40	0.57∼2.31	0.08∼1.88	2.13∼14.02	0.36∼6.14	—	0.38∼2.48	—	—	MS	Deng et al. (2008)
North	0.26∼2.83	0.34∼2.05	0.03∼0.65	1.29∼11.32	2.10∼32.96	—	—	—	—	FLD	Xia et al. (2014a)
North	0.45∼2.92	0.08∼0.90	0.05∼0.97	3.35∼10.59	0.75∼3.72	—	—	—	—	GC-MS	Xia et al. (2014b)
North	0.30∼3.10	0.10∼4.60	2.40	0.10∼9.50	—	—	1.40∼7.80	—	—	UV	Geng and Yang, (2007)
North	0.90∼1.60	2.10∼4.40	0.20∼1.00	5.30∼7.60	1.30∼3.40	—	0.20∼0.40	—	—	UV	Wang et al. (2015)
North	0.97∼1.78	2.06∼3.76	0.27∼0.83	3.93∼6.81	1.40∼2.59	—	0.20∼0.44	—	—	UV	Dou et al. (2014)
North	0.60∼0.80	—	2.20∼2.90	5.70∼6.40	2.00∼2.90	0.30∼0.60	0.04∼0.08	—	—	UV	Avula et al. (2005)
North	0.66∼1.44	0.51∼0.81	0.24∼1.27	3.73∼6.37	1.32∼2.61	—	—	—	—	UV	Lee and Kim, (2010)
North	1.10	3.20	—	6.00	—	—	—	—	—	UV	Tian et al. (2007)
North	0.60∼2.25	0.09∼4.52	0.12∼0.72	0.13∼5.53	0.07∼3.11	—	0.83∼6.14	—	—	UV	Zhang Hai et al. (2009)
North	0.002∼0.235	0.02∼1.79	—	0.47∼5.44	0.17∼1.71	—	—	—	—	UV	Halstead et al. (2007)
North	—	2.50	—	5.00	—	—	—	—	—	UV	Liu et al. (2015)
North	1.60∼4.00	—	—	0.10∼0.70	—	—	0.30∼3.00	—	—	UV	Zhu et al. (2007)
South	3.00∼7.30	0.10∼0.80	0.10∼0.90	0.10∼0.50	0.10∼0.90	—	2.90∼8.30	—	2.10∼5.20	UV	Gao et al. (2003)
South	—	—	—	—	—	—	5.50∼7.70	—	—	UV	Wang et al. (2017)
South	—	—	—	—	—	—	5.38∼5.81	—	—		Gu et al. (2008)
South	1.54∼6.63	<0.21	0.03∼0.89	<0.76	<0.51	—	0.80∼5.66	0.01∼1.48	1.57∼3.82	UV	Wei et al. (2010)
South	1.95∼4.63	<0.15	<0.23	<0.16	<0.42	—	0.37∼3.13	0.11∼0.53	1.02∼2.21	UV	Yuan et al. (2011)
South	—	—	—	—	—	—	0.15∼1.71	0.11∼8.53	0.33∼1.35	MS	Yang et al. (2014)
South	1.90	2.30	5.90	4.80	2.20	—	—	—	—	MS	Song, (2004)

The content results of North Wu Wei Zi (S. chinensis) were listed in descending order according to the maximum value of schisandrin (SCH) content in each literature.

The content results of South Wu Wei Zi (S. sphenanthera) were listed in descending order according to the maximum value of gomisin C (Gom C) content in each literature.

Det. is short for detection method. Abbr. is short for abbreviation.

The major schisandra lignans in North Wu Wei Zi were found to be schisandrin B (Sch B), schisandrin (SCH) and gomisin A (Gom A) (Ren and Zhang, 2020), while South Wu Wei Zi contains higher levels of anwulignan, schisandrin A (Sch A) and gomisin C (Gom C) (Sun et al., 2014). As such, SCH and Gom C are defined as the quality markers of North Wu Wei Zi and South Wu Wei Zi, respectively, according to the Chinese pharmacopoeia, where the SCH content is required to be above 0.40% (North Wu Wei Zi), and Gom C content should be higher than above 0.20% (South Wu Wei Zi).

In addition to species, the place of origin and associated climatic conditions could also affect the lignan contents. It was found that North Wu Wei Zi has a higher enrichment with schisandra lignans when collected from Zhongtiao Mountain, south and east of Qinling mountain, and south of Taihang mountain than from other locations. Similarly, the content of Gom C decreases as rainfall increases, and anwulignan content was reduced by higher maximum temperatures (Sun et al., 2014). It has also been reported that cultivated North Wu Wei Zi that were planted in a north-south orientation had higher lignan accumulation, with SCH, Sch A, and Sch B content in north-south planted North Wu Wei Zi 10.2, 25.8, and 26.8% higher than in those planted in an east-west orientation (Yang, 2011).

## The “ADME” of the Primary Schisandra Lignans

### Absorption

Pharmacokinetic studies have found that the lignans of Wu Wei Zi are readily absorbed into the bloodstream after oral ingestion. Following intragastric (i.g.) administration of Wuzhi capsule (a Chinese patent medicine composed of the ethanol extract of South Wu Wei Zi), Sch A, SCH, Gom A, Gom C, and schisanhenol were rapidly absorbed into the blood and were subsequently metabolized fully before elimination (Wei et al., 2010b; Wei et al., 2014; Wang et al., 2016).

Previous studies showed that SCH, the main lignan in North Wu Wei Zi, could be quickly absorpted after oral administration, with T_max_ less lower than 1 h (Wang et al., 2003; Yan et al., 2006). Further study revealed that it is primarily absorbed in the duodenum and jejunum, followed by the colon and rectum, with absorption percentage of 37.4, 36.1, 24.9 and 31.4%, respectively (Zhang W et al., 2009). In contrast, Sch A and Sch B showed relative slower absorption. For example, the T_max_ of Sch A was found to be between 2 and 4 h after i.g. of South Wu Wei Zi ethanol extract oil in rats (Wei et al., 2010a; Li H. L. et al., 2013), and a secondary absorption could be observed around 8 h (Li WL. et al., 2013). Sch B exhibited the first plasma concentration peak at 5.5–6.0 h, closely followed by a second absorption concentration peak 0.5 h after the first peak. Currently available data showed that the absorption rates are much faster in mice compared with rats, with observed secondary absorption of Sch C, Gom A, Gom B, and Gom C appearing at 8–25 h after North Wu Wei Zi extract administration. Meanwhile, the secondary C_max_ was often lower that the primary one (Wei Hua et al., 2013; Liu et al., 2017). It is suggested that the secondary peak may be caused either by enterohepatic circulation or by the metabolic transformation from other schisandra lignans.

The majority of the schisandra lignans could change their absorption characteristics when Wu Wei Zi was administrated along with other medical materials. For example, the absorption rate of SCH decreased slightly (T_max_ 0.32∼1.00 h) when North Wu Wei Zi was administrated in the prescription “Shengmai Powder” which also contains Radix Ophipogonis and Radix et Rhizoma Ginseng (Wang et al., 2003; Li et al., 2005; Guo et al., 2012). Moreover, SCH showed a significantly decreased T_max_ at 7.0 h when administrated in the prescription “Longlingchun mixture”, which is composed of North Wu Wei Zi and other ten Chinese medicines (Zhao et al., 2006).

### Distribution

After absorption, or after tail vein injection, the highest concentrations of the main schisandra lignans (such as SCH, Sch A, and Sch B) were presented in the lungs and liver, followed by the heart, kidneys, and lastly the spleen (Zhou et al., 2013). Sch A and SCH were also both found in rat brain at a very low concentration level, suggesting that they were able to cross the blood-brain barrier (Hu, 2012). Within the different rat brain regions, SCH showed the highest concentrations in the hypothalamus, striatum, and hippocampus, followed by the brain stem, and the lowest concentration of which was found in the cerebral cortex and cerebellum. The high distribution in hypothalamus, striatum, and hippocampus may explain the neuro-protective effects of SCH and North Wu Wei Zi on mice with Alzheimer’s disease and depression (Wang et al., 2013; Wang Baolian et al., 2014).

### Metabolism

**FIGURE 2:**
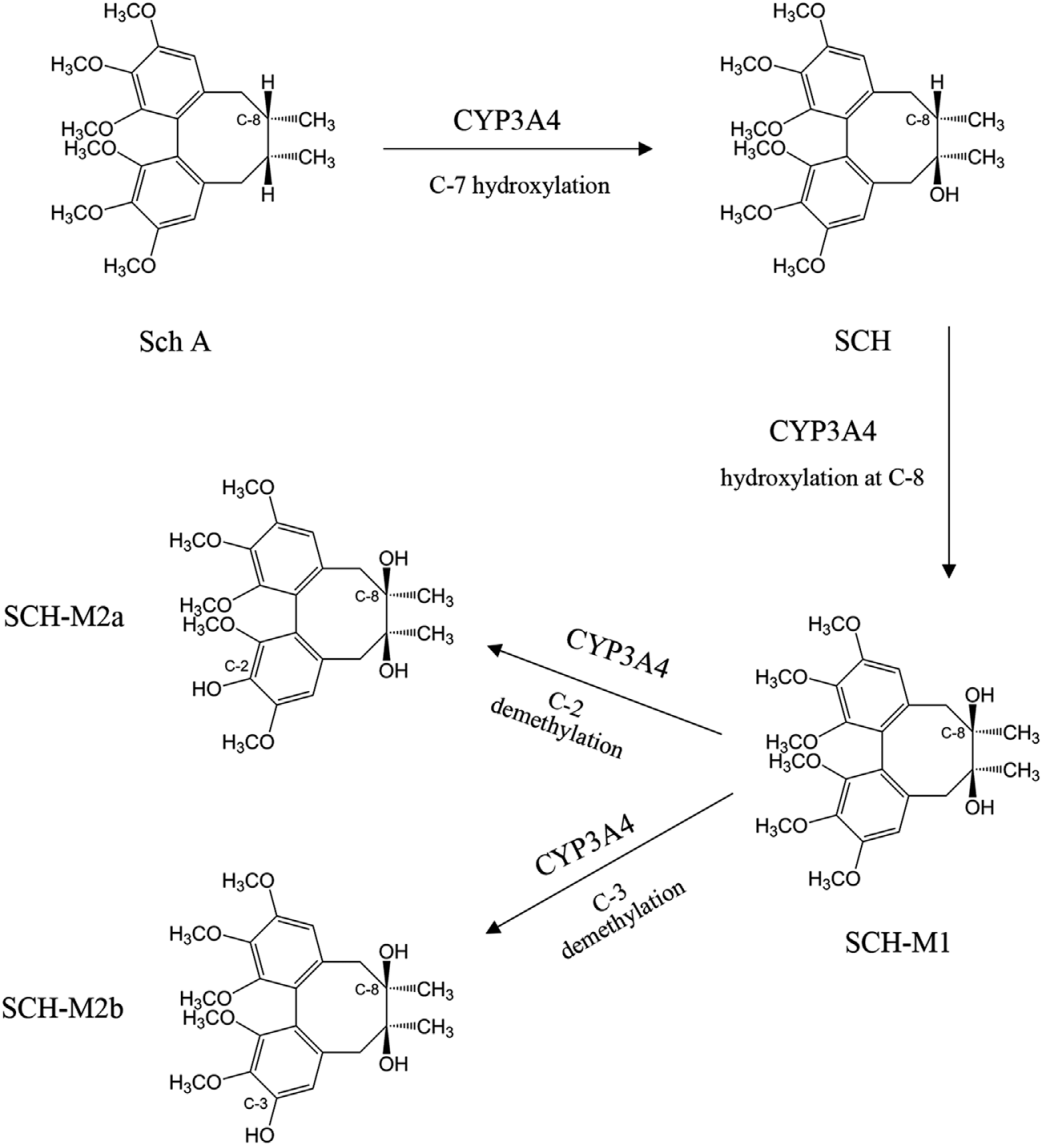
The metabolism of schisandrin A (Sch A) and schisandrin (SCH).

The major phase I metabolism pathways of schisandra lignans are demethylation and hydroxylation (Zhang et al., 2014; Zhang et al., 2017a), including a total of 33 metabolites characterized from six lignans following administration of a South Wu Wei Zi preparation. In detail, the enzyme CYP3A4 catalyzes Sch A to SCH in the first step (Cao, 2010). Secondly, SCH formed SCH-M1, mediated by CYP3A4 again at hydroxylation of C8 position, and then biotransformed by demethylation of C2 position (SCH-M2a) or C3 position (SCH-M2b) (Figure 2). The production of SCH-M2b was characterizes two-thirds of that of SCH-M2a in the rat liver microsomes study. The main metabolic pathways of Sch B have been revealed as the mono-oxygenation of C4 or C11 position, the demethylation of the methoxy group on C12 position, or demethylation at the methylenedioxy group connected with C2 and C3 positions which opens the five-member ring, followed by glucuronidation on C3 position (Qian et al., 2015).

### Elimination

Wu et al. (2014a) reported that the main schisandra lignans SCH, Sch A, and Sch B, were fully eliminated within 25 h in male rats following South Wu Wei Zi extract administration. Basically, schisandra lignans and their metabolites are gradually excreted through feces and urine (Qian et al., 2015).

## Wu Wei Zi Induced Drug-Drug Interactions

### The Biphasic Effect of Schisandra Lignans on CYPs

DDIs have been implicated in some studies for both North Wu Wei Zi and South Wu Wei Zi. Interestingly, recent studies have identified seemingly contradictory inductive and inhibitory effects of their water or ethanol extracts, and more specifically schisandra lignans, on CYPs and P-gp.

Both Wu et al. (2007a) and Mao and Tay (2016) found that North Wu Wei Zi extract inhibited CYP3A in human liver microsomes (HLMs; *in vitro*) and in healthy volunteers (*in vivo*). In contrast, Hu (2011) reported that North Wu Wei Zi extract increased CYP450 contents in Kunming mice after 7-day-oral administration. The bi-directional effect on CYPs was first identified by Cheng et al., and it was found that CYP3A activity in rats was inhibited within 12 h after North Wu Wei Zi water extract administration yet was increased after 6-day-oral administration. The phenomenon was further discussed by Chen et al. (2010), suggesting that the bi-directional effect of North Wu Wei Zi extracts on CYPs depend on the pre- or co-treatment time period. The inhibition effect could be observed immediately after administration, while the induction effect could only be monitored till the episode of DNA transcription and protein synthesis. Besides, a few schisandra lignans showed contradictory effects on CYP activities when involving individually in different experimental conditions. For example, Sch B was identified as a CYP inducer when administrated to mice *in vivo* (Liu, 1988), yet could inhibit CYP3A activity when incubated with rat liver microsomes *in vitro* (Li et al., 2010). In general, both North Wu Wei Zi and South Wu Wei Zi extracts, Sch A, Sch B, and SCH are recognized as activators when administered *in vivo* with long-term administration (>6 days), and as inhibitors when incubated with liver microsomes or CYP isoforms *in vitro* (Tables 2, 3).

**TABLE 2:** Biphasic effect on regulating CYP activities by Wu Wei Zi extract.

Extract	*in vivo*/*in vitro* ^a^	Bi-directional effect on CYPs^b^	IC_50_ (μg/ml)	Dosage/Application	CYP Substrate^d^	Note	Ref.
North Wu Wei Zi water extract	*in vitro*, RLM	CYP1A2 -47%	—	500 μg/ml	PNT-CYP1A2	—	Wang et al. (2011)
		CYP2C6 -53%			DFS-CYP2C6		
		CYP2C11 -48%			MPT-CYP2C11		
		CYP2D2 -40%			DTM-CYP2D2		
		CYP2E1 -57%			CLZ-CYP2E1		
		CYP3A-70%			MDZ-CYP3A		
	*in vitro*, RLM	CYP3A-	487.8	—	TST	—	Chen et al. (2010)
	*in vitro*, RH	CYP3A+55%	—	2.5 mg/ml, 3 days	TST	—	Chen et al. (2010)
	*in vivo*, rat	CYP2D2—CYP2E1 +	—	1.5 g/kg/d, 7 days	DTM-CYP2D2	—	Wang et al. (2011)
				EDH^c^: 13.1 g/d	CLZ-CYP2E1		
	*in vivo*, rat	CYP3A-51∼60%	—	1∼3 g/kg/d, 1 day	TST	—	Chen et al. (2010)
				EDH^d^: 8.6∼25.7 g/d			
	*in vivo*, rat	CYP3A+36∼58%	—	1∼3 g/kg/d, 3 days	TST	—	
				EDH^c^: 8.6∼25.7 g/d			
	*in vivo*, rat	total CYP +	—	3 g/kg/d, 1 day, tested after 6 days	—	mRNA transcription +	Mu et al. (2006)
				EDH^c^, 18.9 g/d			
North Wu Wei Zi ethanol extract	*in vitro*, RLM	CYP1A2 -80%	—	120 μg/ml	PNT-CYP1A2	—	Wang et al. (2011)
		CYP2C6 -90%			DFS-CYP2C6		
		CYP2C11–90%			MPT-CYP2C11		
		CYP2D2 -80%			DTM-CYP2D2		
		CYP2E1 -50%			CLZ-CYP2E1		
		CYP3A-90%			MDZ-CYP3A		
	*in vitro*, HH	CYP3A4 +	—	—	—	mRNA transcription +	Mu et al. (2006)
		CYP2C9 +					
	*in vitro*, MH	CYP3A+	—	—	—	mRNA transcription +	
		CYP2C29 +					
	*in vivo*, rat	CYP2D2 –	—	1.5 g/kg/d, 7 days	MPT-CYP2C11	—	Wang et al. (2011)
		CYP2C11–			DTM-CYP2D2		
		CYP2E1 +		EDH^c^: 13.1 g/d	CLZ-CYP2E1		
		CYP3A+			MDZ-CYP3A		
Schisandra lignan extract of South Wu Wei Zi	*in vitro*, RLM	CYP3A-	40.0 without preincubation	—	MDZ	Mechanism-based inhibition suggested	Lai et al. (2009)
			35.0 with preincubation				
	*in vitro*, RIM	CYP3A-	25 without preincubation	—	MDZ	Mechanism-based inhibition suggested	
			6.3 with preincubation				
	*in vivo*, rat	CYP3A+	—	0.15 g/kg/d, 14 days	MDZ	—	
				EDH^d^: 1.3 g/d			
South Wu Wei Zi ethanol extract	*in vivo*, rat	CYP3A-	—	0.25 g/kg/d, 1 day	paclitaxel	blood exposure +1.9 fold	Jin et al. (2011)
				EDH^c^: 2.1 g/d			

a *In vivo/in vitro*: HH: human hepatocytes; HLM: human liver microsomes; RH: rat hepatocytes; RLM: rat liver microsomes; RIM: rat intestinal microsomes; MH: mouse hepatocytes.

b Bi-directional effect on CYPs: −, inhibition; +, induction.

c EDH: equivalent dosage for human.

d CYP probe substrates: phenacetin (PNT), diclofenac sodium (DFS), mephenytoin (MPT), dextromethorphan (DTM), chlorzoxazone (CLZ), midazolam (MDZ), and testosterone (TST).

**TABLE 3:** Inhibitory of main CYP450 isoform activity by the major schisandra lignans.

Component (abbreviation)	*in vivo*/*in vitro*	Enzyme source^a^	CYP enzyme	Substrate^b^	IC_50_ (μM)	*K*_i_ (μM)	*K*_I_ (μM)	*k*_inact_ (min^−1^)	Inhibition type	Ref.
Schisandrin A (Sch A)	*in vitro*	HLM	CYP3A4	TST	—	1.51	—	—	Reversible, competitive	Zhang et al. (2017b)
	*in vitro*	HLM	CYP3A4	TST	—	74.1 ± 10.1	—	—	Mixed	Wu et al. (2014)
	*in vitro*	RLM	CYP3A	MDZ	70	45	—	—	—	Lai et al. (2009)
	*in vitro*	RLM	CYP3A	MDZ	12.5	4.8	—	—	—	Lai et al. (2009)
	*in vitro*	HLM	CYP2C19	OME	2.77	—	—	—	—	Wei (2010)
	*in vivo*	rat	CYP3A	MDZ	—	30.67 mg/kg	—	—	Reversible, non-competitive	Li Wei-Liang et al. (2012)
	*in vivo*	rat	CYP3A	MDZ	12.5 ± 2.5	30.67	—	—	—	Li Ruidong et al. (2012)
Schisandrin B (Sch B)	*in vitro*	RLM	CYP3A	MDZ	—	4.24	—	—	Reversible, non-competitive	Li et al. (2010)
	*in vitro*	RLM	CYP3A	MDZ	6.25	5	—	—	—	Lai et al. (2009)
	*in vivo*	rat	CYP3A	MDZ	—	16.64 mg/kg	—	—	Reversible, non-competitive	Li WL. et al. (2013)
	*in vitro*	HLM	CYP3A	MDZ, NIF, TST	1.3–4.5	—	—	—	—	Seo et al. (2021)
	*in vitro*	HLM	CYP2C19	DTM	10.4	—	—	—	—	Seo et al. (2021)
	*in vitro*	HLM	CYP2E1	CZX	>50	—	—	—	—	Seo et al. (2021)
Schisandrin C (Sch C)	*in vitro*	HLM	CYP3A	OME	4.01	—	—	—	—	Wei, (2010)
	*in vitro*	HLM	CYP2C19	DTM	2.7	—	—	—	—	Seo et al. (2021)
	*in vitro*	HLM	CYP2E1	CZX	>50					Seo et al. (2021)
Schisandrin (SCH)	*in vitro*	RLM	CYP3A	MDZ	70	45	—	—	—	Lai et al. (2009)
	*in vitro*	HLM	CYP2C19	DTM	5.3	—	—	—	—	Seo et al. (2021)
	*in vitro*	HLM	CYP2E1	CZX	4.2					Seo et al. (2021)
Gomisin A (Gom A)	*in vitro*	RLM	CYP3A	TST			0.35	1.96	irreversible	Zhai et al. (2017)
	*in vitro*	HLM	CYP3A	ERY	10.1	—	—	—	—	Iwata et al. (2004)
	*in vitro*	HLM	CYP3A	TST	6.71	—	—	—	—	Iwata et al. (2004)
	*in vitro*	HLM	CYP3A	MDZ	2.40	—	—	—	—	Wei (2010)
	*in vitro*	HLM	CYP3A	MDZ, NIF, TST	1.8–2.3	—	—	—	—	Seo et al. (2021)
	*in vitro*	HLM	CYP2C9	TBT	3.44	—	—	—	—	Wei (2010)
	*in vitro*	HLM	CYP2C19	OME	5.91	—	—	—	—	Wei (2010)
	*in vitro*	HLM	CYP2C19	DTM	11.2	—	—	—	reversible	Seo et al. (2021)
	*in vitro*	HLM	CYP2D6	DTM	8.58	—	—	—	—	Wei, (2010)
	*in vitro*	HLM	CYP2E1	CZX	>50	—	—	—	reversible	Seo et al. (2021)
Gomisin B (Gom B)	*in vitro*	HLM	CYP3A	ERY	0.399	0.131	—	—	—	Iwata et al. (2004)
	*in vitro*	HLM	CYP3A	TST	0.624	—	—	—	—	Iwata et al. (2004)
	*in vitro*	HLM	CYP3A	MDZ, NIF, TST	0.28–0.42	—	—	—	—	Seo et al. (2021)
	*in vitro*	HLM	CYP2C19	DTM	>50	—	—	—	—	Seo et al. (2021)
	*in vitro*	HLM	CYP2E1	CZX	>50	—	—	—	—	Seo et al. (2021)
Gomisin C (Gom C)	*in vitro*	HLM	CYP3A	ERY	0.254	0.049	0.399	0.092	irreversible	Iwata et al. (2004)
	*in vitro*	HLM	CYP3A	TST	0.257	—	—	—	—	Iwata et al. (2004)
	*in vitro*	RLM	CYP3A	MDZ	0.3	0.06	—	—	—	Lai et al. (2009)
	*in vitro*	HLM	CYP3A	MDZ, NIF, TST	0.19–0.30	—	—	—	—	Seo et al. (2021)
	*in vitro*	HLM	CYP2C19	DTM	16.3	—	—	—	—	Seo et al. (2021)
	*in vitro*	HLM	CYP2E1	CZX	>50	—	—	—	—	Seo et al. (2021)

a RH: rat hepatocytes; HH, human hepatocytes; RLM, rat liver microsomes; HLM, humanliver microsomes.

b CYP probe substrates^b^: testosterone (TST), midazolam (MDZ), omeprazole (OME), nifedipine (NIF), tolbutamide (TBT), dextromethorphan (DTM), and erythromycin (ERY), chlorzoxazone (CZX).

Zhai et al. (2015) refined the theory, suggesting that Wu Wei Zi extracts or their major schisandra lignans exhibit inhibitory or inductive effect on CYPs activities depending on the experimental conditions. Specifically, it is suspected that the main schisandra lignans could induce CYP DNA expression and inhibit CYP protein activity at the same time; however, the CYP inhibition would be observed immediately, explaining CYP-inhibitory effects were only seen in *in vitro* experiments when using liver microsomes or CYP isoforms, which did not rely on DNA transcription and translation. On the other hand, the lignan-induced CYP expression could only occur after DNA transcription and translation had taken place, a process of which apparently could take 5 days or more *in vivo*. The modified theory has been supported by other studies. For example, North Wu Wei Zi water extract inhibited CYP3A activity with single-dose administration and induced CYP3A activity with multiple administration in rats (1–3 g/kg, qd for 3 days) (Chen et al., 2010) (Table 2). This study also showed that the CYP3A induction was caused by an increase of mRNA transcription with Sch B and Gom A.

The inhibition or induction of Wu Wei Zi and its major schisandra lignans is summarized in Tables 2–4. For ease of comparison, the dose of Wu Wei Zi in the *in vivo* studies were converted into human dosage according to an average body weight of 54 kg.

### The Inhibitory Effects of Wu Wei Zi and its Major Lignans on CYP Enzymes

Wu Wei Zi extracts were found with extensive inhibitory effects on CYPs (Table 2). CYP inhibition can generally be classified as reversible or irreversible interactions (Berg et al., 2012), with reversible CYP inhibitions further divided into competitive, non-competitive, and un-competitive interactions according to the interaction between inhibitors and CYP enzymes. For irreversible inhibition, inhibitors are first catalyzed by CYPs into reactive intermediates which then form metabolite-inhibitor complexes with CYP that causes deactivation. As a result, irreversible inhibitions are time-dependent and NADPH-dependent process when the inhibitors exposed to the CYP. This phenomenon could be reinforced by multiple dosing of the inhibitors *in vivo*.

Wang et al. (2011) reported that CYP1A2, CYP2D2, CYP2C6, CYP2C11, CYP2E1, and CYP3A activities could be inhibited by North Wu Wei Zi water/ethanol extract in rat liver microsomes (RLMs). The activities of the CYPs were reduced by 50–90% at a concentration of 120 μg/ml when incubated with North Wu Wei Zi ethanol extract, which was recognized as a stronger CYP inhibitor than North Wu Wei Zi water extract. Moreover, the activities of CYP2D2 and CYP2C11 remained inhibited 1 week after North Wu Wei Zi ethanol administration (i.g., 1.5 g/kg). South Wu Wei Zi was also found to be a potent CYP3A inhibitor in rat hepatic or intestinal microsomes, the lignan extract of which showed IC_50_ values of 25–40 μM. It was further confirmed that Gom C, the most abundant lignan in South Wu Wei Zi, exhibited a strong inhibitory effect in a time-dependent manner, indicating a mechanism-based inhibition (Lai et al., 2009).

Both North and South Wu Wei Zi and their major lignan components have been investigated as CYP inhibitors in DDI studies with clinical-practiced medicines for financial burden reducing, toxicity-alleviating, or pharmacodynamics-enhancing purposes. Take tacrolimus (FK506), a natural immunosuppressant as an example, which is metabolized by CYP3A (Harding et al., 1989; Chen et al., 2020). Its blood concentration can be largely increased by the combination of Wuzhi tablet, a commercially available medicine composed of South Wu Wei Zi ethanol extract, owing to the schisandra lignans’ higher affinity to CYP3A than that of FK506 (Qin et al., 2010a; Qin et al., 2014b; Li et al., 2017; Qin et al., 2020). Thus, Wuzhi tablet is recognized to reduce the oral dosage of FK506 as well as relieve FK506-induced hepatotoxicity, which is similar to Wuzhi capsule (Wei et al., 2014; Wang et al., 2016; Cheng et al., 2021). Many clinical practices have evidenced that both Wuzhi tablet and Wuzhi capsule could effectively increase the blood exposure of FK506 in renal, heart and liver transplanted patients (Jiang et al., 2010; Xin et al., 2011; Liu et al., 2012; Zhou et al., 2017). Similarly, as csyclosporin A was a substrate of CYP3A and P-gp, Wuzhi Tablet could also dramatically increase its C_max_ and the blood exposure of by 84.1 and 293.1%, respectively (Xue et al., 2013). What’s more, Wuzhi tablet was also effective in increasing the blood exposure of paclitaxel by 1.9˗fold in rats, while Gom A could effectively increase the C_max_ of oral-administered paclitaxel by 2.8˗fold at an oral dose of 25 mg/kg (Jin et al., 2011). Meanwhile, the North Wu Wei ethyl acetate extract was found to block the CYP3A-mediated toxic metabolic pathway of cyclophosphamide, and thus reduce the metabolite related liver, kidney, and brain toxicity in rats (Zhai et al., 2018).

With further investigation, it was found that the CYP-inhibitory efficacy comes from several major lignans of south and north Wu Wei Zi. Zhai et al. have identified Gom A as an efficient CYP3A blocker (*K*_I_ = 0.35 µM, *k*_inact_ = 1.96 min^−1^) (Zhai et al., 2017), while Iwata et al. reported that the CYP3A-inhibiting efficacy of Gom C was found stronger than that of ketoconazole (Iwata et al., 2004). Additionally, other schisandra lignans also take roles as CYP inhibitors (Table 3). For example, Sch A and Sch B have both been found to decrease CYP3A activity with IC_50_ ranging from 6 to 70 μM (Li Ruidong et al., 2012; Wu et al., 2014), and the inhibition effect was not reversed by activation after 3 days of continuous administration (Li Wei-Liang et al., 2012; Li WL. et al., 2013).

So far, the major schisandra lignans were found working as CYP-inhibitiors with different mechanisms. The *in vivo* evidences reported that Sch A and B could dose-dependently inhibited hepatic microsomal CYP3A activity as noncompetitive inhibitors (*K*i _Sch A_ = 30.67 mg/kg; *K*i _Sch B_ = 16.64 mg/kg) (Li Ruidong et al., 2012; Li H. L. et al., 2013). Meanwhile, Gom A was found as a mix-type CYP3A inhibitor, which showed characteristics of competitive as well as time- and NADPH- dependent inhibition (Zhai et al., 2017), while Gom C could irreversibly inactivate CYP3A by forming metabolite-inhibitor complexes (Iwata et al., 2004). It is suspected that the potent inhibition on CYP3A may be caused by the metabolite-intermediate complexes formed by the methylenedioxyphenyl structure with P450 enzyme (Iwata et al., 2004; Lai et al., 2009; Seo et al., 2021). In all, lignans with one methylenedioxyphenyl group such as Gom A, Gom B, Gom C, and Sch B most strongly inhibited CYP3A activity, with IC_50_ values as low as 0.19–0.28 μM, which was much lower than those of the other P450 isoforms. For these schisandra lignans, some researches obtained larger IC_50_ values, the discrepancy of which could be due to differences in incubation conditions including CYP3A probe substrates and/or the enzyme source. Among those schisandra lignans, most of them showed potent or moderate inhibition of on CYP2C19, with IC_50_ values ≤16.3 μM, except Gom B (>50 μM). Sch C with two methylenedioxyphenyl groups showed most inhibitory effect on CYP2C19, with a IC_50_ value of 2.7 μM. Only SCH, containing none methylenedioxyphenyl group but one hydroxyl group, moderately inhibited CYP2E1(IC_50_ value 4.2 μM).

### The Inductive Effects of Wu Wei Zi and its Major Lignans on CYP Enzyme Activity

Since the late 1960s, both North Wu Wei Zi and South Wu Wei Zi extracts have shown hepatic-protective effects in clinical practice (Zuo et al., 2019). Apart from the alleviation of inflammatory response, it was found that North Wu Wei Zi extract significantly increased the smooth endoplasmic reticulum in hepatocytes of rats after continuous ingestion (≥3 days, qd), which was consistent with the increased liver CYP content and the metabolism acceleration of toxic components in experimental animals (Qiu et al., 2018). More studies found that multiple prolonged administration of Wu Wei Zi extract (≥3 days, qd) could lead to CYP induction, increasing activities of CYP3A, CYP2C, and CYP2E (Chen et al., 2010; Mu et al., 2006) (Table 4), and its administration at high dose could be more likely to cause CYP induction (≥1 g/kg/d for rats, which is equivalent to≥8.6 g/d for human, Table 2). Besides, continuous ingestion of Wu Wei Zi extract at low dose (0.15 g/kg/d for rats, which is equivalent to 1.3 g/d for human) for 2 weeks was also found to cause CYP3A induction in rats (Lai et al., 2009).

**TABLE 4:** Induction of CYP3A activity by the major schisandra lignans.

Component (Synonym)	*in vitro/in vivo* ^a^	Induction	Substrate^b^	Dosage/Application^d^	Increased	Ref.
		On CYPs				
Schisandrin A (deoxyschisandrin)	*in vitro*, HH	CYP3A4 +	—	—	mRNA transcription	Mu et al. (2006)
Schisandrin B (*γ*-schisandrin)	*in vitro*, HH	CYP3A4 +	—	—	mRNA transcription	Mu et al. (2006)
	*in vitro*, RH	CYP3A+21∼42%	TST	0.01∼0.1 µM, 3 days incubation	mRNA transcription	Chen et al. (2010)
	*in vivo*, rat	CYP3A	MDZ	—	—	Li H.L. et al. (2013)
Gomisin A (schisandrol B)	*in vitro*, HH	CYP3A4 +	—	—	mRNA transcription	Mu et al. (2006)
	*in vitro*, RH	CYP3A+27%	TST	10 µM	mRNA transcription	Chen et al. (2010)
	*in vivo*, rat	CYP3A-	paclitaxel	25 mg/kg/d, 1 day	2.8-fold blood exposure	Jin et al. (2010)
				EDH^c^: 214 mg/d		

a *In vitro/in vivo*: RH: rat hepatocytes; HH, human hepatocytes.

b CYP probe substrates: midazolam (MDZ); testosterone (TST).

c EDH: equivalent dosage for human.

Mu et al. (2006) further suggested that the mechanism of CYP induction of Wu Wei Zi extract may be caused by the activation of the pregnane X receptor, with Sch A, Sch B, and Gom B as major responsible agonists. Interestingly, recent investigations revealed that pregnane X receptor might also be the key target for the hepato-protective efficacy of Gom A. By activating the pregnane X receptor, Gom A could accelerate the bile acid metabolism, promote bile acid efflux and induce hepatic expression of Cyp3a in mice (Zeng et al., 2017).

### Effects of Wu Wei Zi and Their Major Lignans on P-Glycoprotein Activity

As well as the CYP-dependent metabolism changes, phytochemical-mediated alterations in P-glycoprotein (P-gp) activity may also produce DDIs by altering drug absorption, distribution, and elimination, which could be achieved either by decreasing P-gp expression or by inhibiting P-gp activity. For example, North Wu Wei Zi extract could effectively increase the blood exposure of P-gp substrate taninolol by 47% in healthy volunteers (Fan et al., 2009). A number of studies have shown that Gom A, Gom C, SCH, Sch A, and Sch B could reverse P-gp-mediated multidrug resistance (MDR) and decrease the efflux of P-gp substrate in cancer cells (Wan et al., 2006; Fong et al., 2007; Li et al., 2007; Yoo et al., 2007; Qin et al., 2010b).

Interestingly, a subsequent transport study using Caco-2 cells also found that Gom A, Gom C, Sch A, and SCH were not substrates of P-gp (Qin et al., 2014a). It was found that instead of directly inhibiting P-gp activity, the underlying mechanism of the schisandra lignans may be intervening with the substrate-P-gp complex and blocking the active P-gp transport sites (Fong et al., 2007). What’s more, continuous treatment with schisandra lignan extract for 10 days (or >10 days) would cause massive P-gp expression decrease in rat intestinal and brain tissues (Liang et al., 2013).

## Discussion and Conclusion

In clinical setting, combination with traditional Chinese medicine is usually used to enhance the efficacy and/or reduce adverse reactions of modern medicines. Over the past few years, there have been many reports concerning the pharmacokinetic interactions involving the components of Wu Wei Zi in the form of herb extract, herb decoction, or herb preparation (Feng et al., 2017). Therefore, Table 5 summarized some DDI in animal experiments and clinical researches. It was found that Wu Wei Zi and its preparations have the capacity of affecting the blood concentration/exposure and pharmacokinetic profiles of other drugs. For example, the blood concentration of FK506 *in vivo* mostly contributed to the inhibition of CYP3A4 and/or P-gp via schisandra lignans when co-administration both in single and multiple doses. Meanwhile, the blood concentrations of the schisandra lignans were decreased because of their higher affinity to CYP3A. The schisandra lignans could also enhance the blood exposure of the compounds which were metabolized by CYP2D6 and CYP2D2, or transported by P-gp. The same compound showed different increases in the C_max_ or AUC when the schisandra lignans co-administrated in different situation, indicating the occurrence of DDI was due to be the overall effect of all of the complex chemical components rather than the activity of an individual component. Considering the similarity of the chemical structure of the major schisandra lignans and their transformation during the episode of their metabolism, it is possible that the CYP3A/P-gp inhibitory activity of Wu Wei Zi should be considered as an overall and comprehensive effect of all schisandra lignans and their metabolites. It is important to point out that although schisandra lignans exhibit the potential for other CYPs-associated DDI based mostly on *in vitro* experimental results (Tables 2-4), “clinically relevant” DDI mediated by these CYPs (such as 2C19 and CYP2E1) are limited.

**TABLE 5:** Reported DDIs in animal experiments and clinical researches.

Subjects	Drug dosage and administration route	Some key pharmacokinetic parameters	Underlying mechanism	Ref.
SD male rats	Wuzhi Tablet: 0.25 g/kg, i.g., once	FK506: C_max_ +80.1%, AUC_0−t_ +2.1-fold	The inhibition of FK506 metabolism mostly contributed to the increase of oral FK506 exposure for the interplay of CYP3A and P-gp in liver by Wuzhi Tablet.	Qin et al. (2010a)
	FK506: 1.89 mg/kg, i.g., once			
SD male rats	Wuzhi tablet: 250 mg/kg, i.g., once	FK506: Cmax +1.64-fold	Wuzhi tablet could enhance the blood concentration of FK506 *in vivo*, which might be due to inhibition of CYP3A4 and/or P-gp via substances	Qin et al. (2010b)
	FK506: 3.78 mg/kg/day, i.g., once			
SD male rats	Schisandra lignans (Sch A, Sch B, Sch C, SCH, Gom A, and Gom C): 0.024 mg/kg, i.g., once	FK506: AUC_0-t_ +152.0%, +109.6%, +46.4%, +41.4%, +598.4%, and +159.5%, by a single oral dose co-administration of Sch A, Sch B, Sch C, SCH, Gom A, and Gom C, respectively	The exposure of FK506 in rats was increased when co-administered with the lignans, which inhibited P-gp–mediated efflux and CYP3A-mediated metabolism of FK506, and reduced the intestinal first-pass action	Qin et al. (2014a)
	FK506: 1.89 mg/kg, i.g., once			
SD male rats	Wuzhi tablet: 0.25 g/kg, i.g., once FK506: 1.89 mg/kg, i.g., once	With FK506 co-administration	The blood concentrations of the lignans were decreased and their CYP3A-mediated metabolisms were increased in the presence of FK506 since these lignans had higher affinity to CYP3A	Qin et al. (2014b)
		Sch A: AUC_0-t_ -47.2%	It was suggested that the lignans have higher affinity to CYP3A, so that their blood concentrations were decreased while the CYP3A-mediated metabolisms were increased in the presence of FK506. Transport study in Caco-2 cells showed that these lignans were not substrates of P-gp, suggesting decreased blood concentration of lignans by FK506 was not via P-gp pathway	
		SCH: AUC_0-t_ -55.1%		
		Gom A: AUC_0-t_ -57.4%		
		Gom C: AUC_0-t_ -64.5%		
SD male rats	Wuzhi tablet: 62.5∼750 mg/kg, i.g., once	FK506: AUC_0-t_ +1.76, +1.26, +2.48, +0.97, +0.87 and +0.36-fold, respectively, with Wuzhi tablet pretreatment (0, 0.5, 2, 6, 12 and 24 h before FK506 administration, 250 mg/kg)	The CYP3A activity was irreversibly inactivated by Wuzhi tablet, although the mRNA and protein expression of CYP3A was significantly induced after the long-term WZ treatment	Qin et al. (2020)
	FK506: 1.89 mg/kg, i.g., once	FK506: AUC_0-t_ +0.07, +0.44, +1.60, +1.32 and +1.42-fold, respectively, with Wuzhi tablet co-administration (62.5, 125, 250, 500 or 750 mg/kg)		
SD male rats	Wuzhi capsule: 0.25 g/kg/day, i.g., once	FK506: AUC_0–24 h_ + 128%, clearance -68%	The increase in blood FK506 concentration is caused by the strong inhibition of Wuzhi capsule on P-gp-mediated efflux and CYP3A metabolism	Qin et al. (2013)
	FK506: 1.89 mg/kg, i.g., once			
SD male rats	Wuzhi capsule: 450 mg/kg, i.g., once and qd×12 days	FK506: C_max_ +5.0-fold, AUC_0-∞_+4.9-fold by single dose of Wuzhi capsule	By inhibiting CYP3A and P-gp activity, the reduction of intestinal first-pass effect of FK506 is extensive and contributes greatly to the increase in FK506 bioavailability	Wei Bibbin et al. (2013)
	FK506:1.2 mg/kg, i.g., once	FK506: C_max_ +1.17-fold, AUC_0-∞_+1.29-foldby consecutive 12-day Wuzhi capsule pretreatment		
SD male rats	North Wu Wei Zi water extract 1.5 g/kg/day, i.g., qd×7 days	By North Wu Wei Zi ethanol extract pretreatment	Multiple administrations of North Wu Wei Zi water extract increased the activities, mRNA and protein expressions of CYP2E1, and meanwhile, inhibited the activities and mRNA expression of CYP2D2 *in vivo*. The multiple administration might also inhibit the activity or expression of P-gp, which prevailed over CYP3A induction of and resulted in the elevation of FK506 plasma concentration	Wang Baolian et al. (2014)
	North Wu Wei Zi ethanol extract 1.5 g/kg/day, i.g., qd×7 days	FK506: C_max_ +9-fold, AUC_0-t_ +6-fold		
	FK506, 1 mg/kg, i.g.., once	Chlorzoxazone: AUC_0-t_ -35%, Cmax -20%		
	Chlorzoxazone, 100 mg/kg, i.g., once	Sertraline: Cmax +30%		
	Sertraline, 20 mg/kg, i.g., once			
SD male rats	Wuzhi capsule: 0.25 g/kg/day, i.g., once	Paclitaxel, i.g.: C_max_ +95%, AUC_0-∞_+94%	Inhibition of the activity of P-gp and/or cytochrome P450 enzymes may contribute to the decrease of clearance of paclitaxel	Jin et al. (2011)
	Paclitaxel: 30 mg/kg, i.g., once; or 0.5 mg/kg, i.v., once	Paclitaxel, i.v.: AUC_0-∞_+ 30%		
SD male rats	Wuzhi capsule: 0.25 g/kg/day, i.g., once	Cyclosporine A (37.8 mg/kg): C_max_ +13.1%, AUC_0-∞_+40.1%	That cyclosporine A concentration is slightly increased in the presence of concomitant Wuzhi capsule due to inhibition of CYP3A4 and/or P-gp	Xue et al. (2013)
	Cyclosporin A: 37.8 or 1.89 mg/kg, i.g., once	Cyclosporine A (1.89 mg/kg): C_max_ +84.1%, AUC_0-∞_+2.9-fold		
SD male rats	North Wu Wei Zi ethanol extract: 54, 108, 216 mg/kg, i.g., once	Cyclosporine A: C_max_ +26%, AUC_0−t_ +2-fold	The increase in systemic exposure of cyclosporine A was probably caused by the strong inhibition of North Wu Wei Zi ethanol extract on P-gp-mediated efflux and CYP3A-mediated metabolism of cyclosporine A	Lai et al. (2015)
	Cyclosporine A: 25 mg/kg, i.g., once			
SD male rats	North Wu Wei Zi water extract 0.75 g/kg/day, i.g., once and qd×7 days	By North Wu Wei Zi water extract pretreatment	Inhibition on liver microsomal CYP3A *in vivo*. with multiple administration	Zhai et al. (2018)
	Cyclophosphamide: 300 mg/kg, i.v., once	DCCTX (Cyclophosphamide metabolite)		
		C_max_ -69%, AUC_0-t_ -49% when North Wu Wei Zi was administrated once		
		C_max_ -25%, AUC_0-t_ -37% when North Wu Wei Zi was administrated by consecutive 7-day		
SD male rats	Wuzhi capsule: 300 mg/kg/day, i.g., once	DCCTX: C_max_ -33.10%, AUC_0-t_ -35.51%	The decreased DCCTX concentration was attributed by CYP3A inhibition effect of Wuzhi capsule	Chen et al. (2019)
	Cyclophosphamide: 300 mg/kg, i.v			
SD male rats	Wuzhi capsule: 450 mg/kg, i.g., once and qd×7 days	Methotrexate: C_max_ +1.18-fold, AUC_0-t_ +79% with single dose of Wuzhi capsule	The inhibition of OAT1/3 and P-gp expression by Wuzhi capsule led to decrease in the clearance and increase in the blood exposure of methotrexate	Fu et al. (2021)
	Methotrexate: 2 mg/kg, i.g., once	Methotrexate: C_max_ +1.53-fold, AUC_0-t_ +61% by consecutive 7-day North Wu Wei Zi ethanol extract pretreatment		
SD male rats	Wuzhi capsule: 450 mg/kg, i.g., qd×7 days	With Wuzhi capsule pretreatment	The increase in blood exposure was caused by the P-gp inhibition in intestine, along with the inhibition of metabolic enzymes	Cui et al. (2021)
	Lenvatinib:1.2 mg/kg, i.g., once	Lenvatinib: C_max_ +1.69-fold, AUC_0−t_ +62.7%		
SD male rats	North Wu Wei Zi: 3, 10 g/kg, i.g., once	North Wu Wei Zi extract did not significantly alter the pharmacokinetics of lamivudine	A possible reason is that lamivudine is mainly excreted through the kidney and is not significantly metabolized by CYPs	Li et al. (2020)
	Lamivudine: 10 mg/kg, i.v., once			
SD male rats	North Wu Wei Zi: 500 mg/kg, i.g., once	Warfarin: AUC_0-∞_ -29%	Wu Wei Zi activated PXR and induced CYP3As and 2Cs and then increased warfarin metabolism	Mu et al. (2006)
	Warfarin, 2 mg/kg, i.v., once			
SD male rats	DZSM (containing 20% North Wu Wei Zi): 97.2 mg/kg, i.g., once Clopidogrel: 6.75 mg/kg, i.g., once	Clopidogrel: AUC_c_ +1.25-fold, C_max_ + 18%	DZSM extract inhibited clopidogrel metabolism in rat liver microsomes in a dose-dependent manner	Chen et al. (2016)
		Clopidogrel metabolite: C_max_ -65.8%, AUC_0-∞_ -64.8% IC_50_ of DZSM extract against clopidogrel metabolism is 0.02 mg/ml		
SD female rats	Schisandra lignan extract of North Wu Wei Zi: 500 mg/kg, i.g., once and qd×10 days	With Schisandra lignan extract single-dose pretreatment	Schisandra lignan extract may enhance the exposure of ginsenosides through inhibiting the activity and expression of P-gp	Liang et al. (2014)
	Digoxin: 0.5 mg/kg, i.g., once	Digoxin: AUC_0−∞t_ +1.25-fold; ginsenoside Rb2: AUC_0−∞_+2.18-fold		
	Ginsenosides extract: 120 mg/kg, i.g., once	ginsenoside Rc: AUC_0−∞_+1.49-fold		
		ginsenoside Rd: AUC_0−∞_ +1.86-fold		
		ginsenoside Rb1: AUC_0−∞_ +1.29-fold		
		With Schisandra lignan extract multi-dose pretreatment (qd×10 days)		
		Digoxin: AUC_0−∞t_ +2.00-fold; ginsenoside Rb2: AUC_0−∞_ +1.33-fold		
		ginsenoside Rc: AUC_0−∞_ +1.49-fold		
		ginsenoside Rd: AUC_0−∞_ +1.60-fold		
		ginsenoside Rb1: AUC_0−∞_ +1.82-fold		
SD male rats	ginsenoside Rg1: 10 mg/kg, i.g., once	Ginsenoside Rg1: AUC_c_ + 3.61-fold; ginsenoside Rb1: AUC_c_ + 1.17-fold	Inhibition on liver microsomal CYP3A4 and CYP2D6 *in vivo*	Zhan et al. (2014)
	ginsenoside Rb1: 10 mg/kg, i.g., once	schisandrin: AUC_c_ + 86%		
	schisandrin: 10 mg/kg, i.g., once	compared with administrated alone		
	i.g. separately or in combination			
Healthy adults (*n* = 18, male and female)	Wuzhi capsule: 33.75 mg/d, p.o. bid×13d	Rapamycin: C_max_ +96.3%, AUC_0−∞_ +106.8%	The variability of rapamycin pharmacokinetic parameters by Wuzhi capsule is probably due to the inhibition of CYP3A and (or) P-gp and (or) intestinal enzymes, resulting in increased absorption and decreased gut metabolism and increased rapamycin bioavailability	Li Wei-Liang et al. (2012)
	Rapamycin: 2 mg, p.o., once			
Healthy male volunteers (*n* = 12, male)	Wuzhi capsule: 33.75 mg/d, p.o. bid×13d	FK506: C_max_ + 227.1%, AUC_0−t_ +164.2%	The increased systemic exposure of FK506 induced by North Wu Wei Zi ethanol extract could be due to inhibition of CYP3A4 and/or P-gp in the intestine, resulting in increased absorption and decreased gut metabolism	Xin et al. (2007)
	FK506: 2 mg, p.o., once			
Renal transplant recipients (*n* = 64, male and female)	Wuzhi capsule: 22.5 mg/d, p.o	The blood trough concentration/dosage of FK506 in the Wuzhi capsule group was significantly higher than the non-Wuzhi capsule group at each time point from1 month to 1 year	Not mentioned	Cheng et al. (2021)
	FK506: 3.0–5.0 mg/d, bid			
Liver transplant recipients (*n* = 46, male and female)	Patients were initially administered FK506 (first phase) and then South Wu Wei Zi extract was provided (second phase)	FK506: C_max_ + 183%, AUC_0-12_ + 212%, with co-administration of South Wu Wei Zi extract	Not mentioned	Jiang et al. (2010)
Renal transplant recipients (*n* = 64, male and female)	Wuzhi capsule, 11.25 mg/d, p.o., bid×6 months	Following treatment with Wuzhi capsule for 6 months, the dosage of FK506 in Wuzhi capsule-treated patients had decreased by 34.0%, and the blood trough concentration had risen by 100.5%	Wuzhi capsule inhibited P-gp-mediated efflux and CYP3A-mediated metabolism of FK506 and that the reduction of the intestinal first-pass effect by Wuzhi capsule was the major cause of the increased FK506 oral bioavailability	Xin et al. (2011)
	FK506: 1.42∼2.05 mg/d, p.o., once			

AUCc: (X0-single/X0-mix)*AUC0-t. X0-single, the dose of single compound administration; X0-mix, the relative dose of each compound in co-administration mixture.

AUC0−t (the area under the curve of 0 to t hours) and AUC0-∞ (the area under the curve of 0 to infinity).

Nowadays, a few *in vitro* studies have evaluated the modulatory effects of individual schisandra lignans on CYP3A/P-gp activities, the interactions and synergistic impact of schisandra lignans on CYP3A/P-gp activities are still remains to be further investigated. Besides, the discovery of a family of nuclear receptors such as pregnane X receptor (PXR), constitutive androstane receptor (CAR) and glucocorticoid receptor (GR) had given insight into the molecular explanation of CYP3A induction by xenobiotics (Qin and Wang, 2019). Regarding that Sch A, Sch B, SCH and Gom C could activate human PXR and induce the CYP3A4 reporter gene assay (Zhang et al., 2020), whether the same phenomenon could be observed for CAR and GR was not known. Therefore, studies should begin to explore the potential of these nuclear receptors as targets for those schisandra lignans to elucidate the ability to modulate the expression of CYP/P-gp whether they directly or through the transcriptional activation of nuclear receptors.

According to “Pharmacopoeia of the People’s Republic of China”, the suggested daily dosage of Wu Wei Zi is 2∼6 g. Based on the above discussion, the dosage is unlikely to affect the CYP activity according to Table 2 (the equivalent CYP inhibitory/inductive dosage of North- and South-Wu Wei Zi extract for human were annotated by “EDH”). Even so, the risk of CYP inhibition is high when patients are taking other CYP-blocking chemical drugs at the same time (especially CYP3A), such as nefazolone, ketoconazole, erythromycin and clarithromycin (Manikandan and Nagini, 2018). On the other hand, long-term use of Wu Wei Zi along with CYP-inducers (such as nevirapine and rifampin) could cause significant CYP induction (Finkelstein et al., 2010). In the above cases, the clinicians should put extra caution in applying drugs with narrow theraputic windows, like theophylline, tizanidine, warfarin, phenytoin, tacrolimus and quinidine (Schutte-Nutgen et al., 2018; Patocka et al., 2020). Meanwhile, clinicians must be aware of the contents and pharmacokinetic features of the schisandra lignans in Wu Wei Zi, which is helpful to understand the potential change of chemical drug efficacy with Wu Wei Zi co-administration, as well as the opposite after discontinuation of Wu Wei Zi. Therefore, this paper aimed to provide enough published data about DDIs induced by Wu Wei Zi and the schisandra lignans, and expected to provide guidance for rational use of chemical drugs or herbal medicines in order to avoid the occurrence of adverse side effects via CYP3A or P-gp inhibition and/or induction. Of note, the information and knowledge that were given in this review were the present practical methods and rules for screening and characterization of CYP3A/P-gp inhibitors and inductors and for facilitating the investigations on CYP3A/P-gp mediated DDI interactions.
